# *N*,*N*′-Bis(2,6-diiso­propyl­phen­yl)-*N*-hy­droxy­formamidine–*N*,*N*′-bis­(2,6-diiso­propyl­phen­yl)-*N*-oxidoformamidinium (1/1)

**DOI:** 10.1107/S2414314626008059

**Published:** 2026-08-07

**Authors:** David O. Juma, Adesola A. Adeleke, Sizwe J. Zamisa, Bernard Omondi, Unathi Bongoza, Eric M. Njogu

**Affiliations:** a School of Agriculture and Science, Discipline of Chemistry, University of KwaZulu-Natal, Private Bag X54001, Durban, 4000, Republic of South Africa; b Multimedia University of Kenya, PO Box 15653-00503, Nairobi, Kenya; Universitat de les Illes Balears, Palma de Mallorca, Spain

**Keywords:** crystal structure, *N*-hy­droxy­formimidamides, zwitterionic and neutral mol­ecules

## Abstract

The title compound, C_25_H_36_N_2_O, crystallized with two independent mol­ecules in the asymmetric unit each with statistical proton disorder within the formamidine backbone, thereby indicating the coexistence of zwitterionic and neutral tautomers. The mol­ecular conformation is described by the dihedral angles between each phenyl ring plane and the formamidine backbone, which are 79.8 (3) and 76.0 (3)°, and by the dihedral angle between the two phenyl ring planes, which is 59.61 (11)°. In the crystal, O—H⋯O and N—H⋯N hydrogen bonds connect the neutral and zwitterionic mol­ecules into cyclic dimers. The methyl groups of one isopropyl residue are statistically disordered.

## Abstract 2

The title compound is a symmetrical di­aryl­formamidine derivative bearing diisopropyl substituents. The compound contains two independent mol­ecules in the asymmetric unit, one being the zwitterionic and the other the neutral forms of the di­aryl­formamidine derivative. The two mol­ecules are connected by O—H⋯O and N—H⋯N hydrogen-bonding inter­actions within a cyclic assembly.

## Graphical abstract

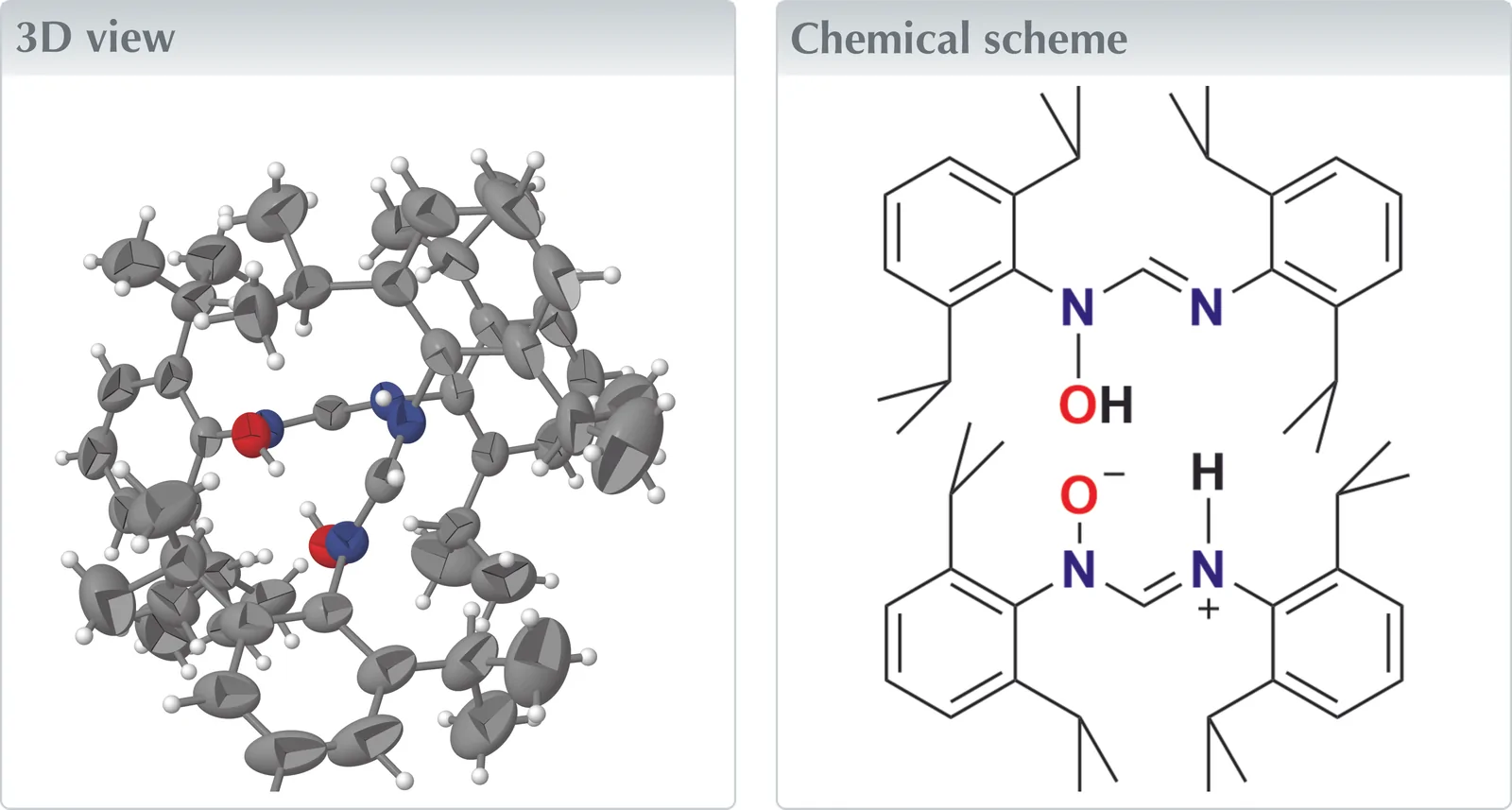

## Structure description

Formimidamide ligands are a distinct and versatile class of nitro­gen-based ligands notable for the range of coordination modes to metal centres (Deacon *et al.*, 2017 ▸). Extensive research has shown that they play significant roles in catalytic processes (Edor *et al.*, 2025 ▸) and in bioinorganic systems (Soliman *et al.*, 2019 ▸; Bongoza *et al.*, 2022 ▸), where their ability to stabilize reactive metal centres is crucial for facilitating catalytic transformations and mimicking enzymatic active sites. A significant subclass of these ligands includes symmetrical di­aryl­formamidine derivatives bearing *N*-hy­droxy­formimidamide functional groups. The *N*-hy­droxy­formimidamides are recognized for their significant role in coordination chemistry, attributed to the hy­droxy substitution on the nitro­gen atom, which influences both electronic characteristics and coordination modes. These mol­ecules can exist in both zwitterionic and neutral forms, impacting their reactivity and coordination properties thereby broadening their potential applications. Moreover, the presence of both tautomeric forms can cause subtle changes in the chemical environments or substituent patterns, influencing stability and inter­molecular inter­actions (Juma *et al.*, 2025 ▸). Recognizing the crucial role of *N*-hy­droxy­formimidamides in coordination chemistry, our research efforts focused on the synthesis and structural elucidation of di­aryl­formamidine derivatives bearing sterically demanding substituents. As such, we herein report the crystal structure of the title compound, providing insight into its mol­ecular conformation and inter­molecular inter­actions.

**Figure 1:**
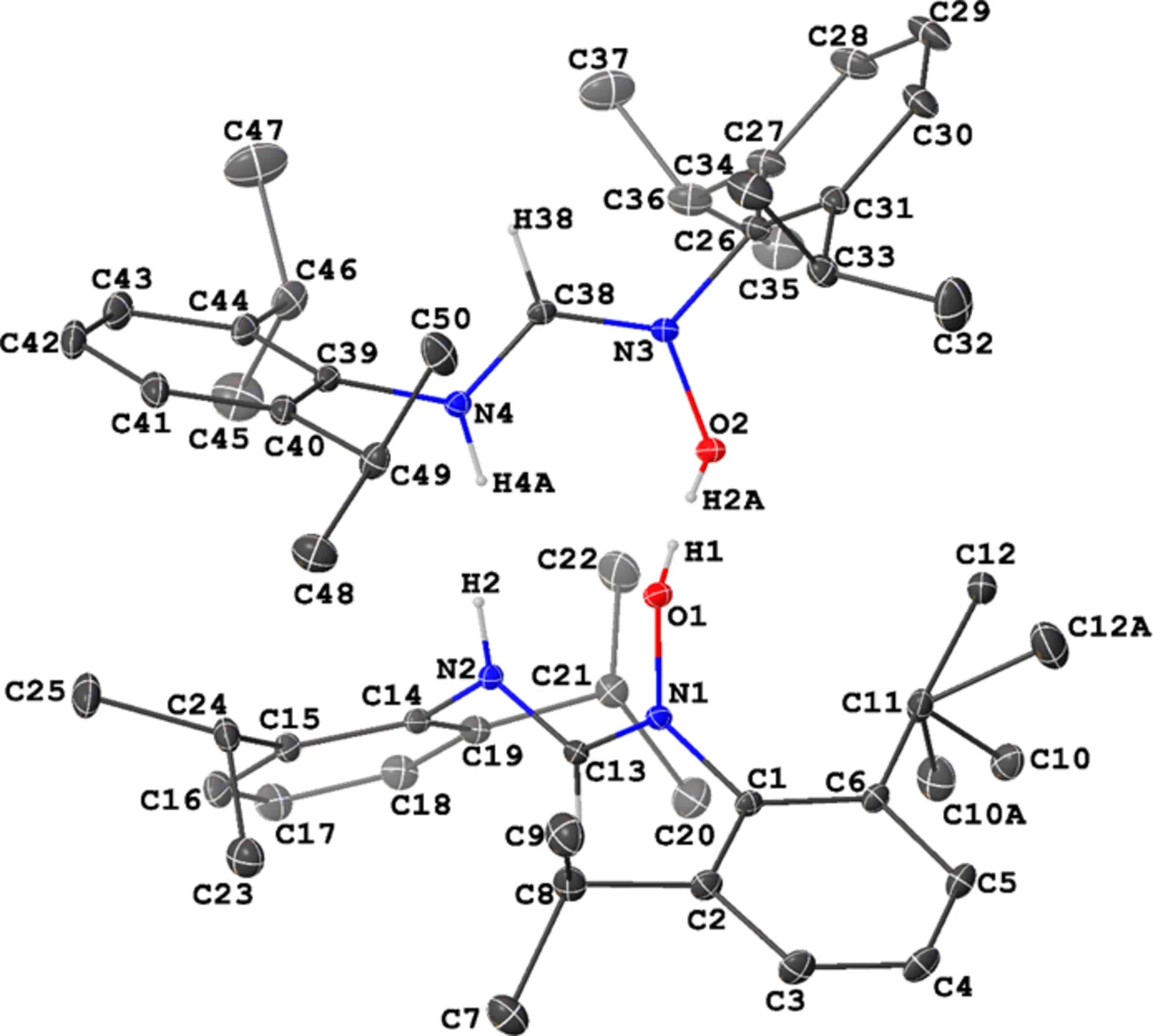
Mol­ecular structures comprising the asymmetric unit of the title compound, shown with displacement ellipsoids at the 50% probability level. Hydrogen atoms, except those on the formamidine backbone, are omitted for clarity. The C10, C12 and the hydrogen atoms on the amine and hy­droxy groups are statistically disordered.

The title compound is a symmetrical di­aryl­formamidine derivative bearing diisopropyl substituents. Its crystal contains two independent mol­ecules in the asymmetric-unit, Fig. 1 ▸. The compound can exist in both zwitterionic and neutral forms (Juma *et al.*, 2025 ▸), as evidenced herein by the disorder of the formamidine hydrogen atoms, each refined with 0.5 site occupancy over N2/N4 and O1/O2. The mol­ecular conformation is described by the mean dihedral angles (averaged over the two independent mol­ecules in the asymmetric unit) between each phenyl-ring plane and the formamidine backbone [–N(OH)–C(H)=N–]. The phenyl ring attached to the *N*-hy­droxy group exhibits a mean dihedral angle of 79.8 (3)°, while the phenyl ring bound to the amine nitro­gen shows a mean dihedral angle of 76.0 (3)°. The mean dihedral angle between the planes of the two phenyl rings is 59.61 (11)°. All other intra­molecular bond parameters are comparable with those of *N*^1^,*N*^2^-bis­(2,6- di­methyl­phen­yl)-*N*^1^-hy­droxy­formamidine *N*,*N′*-bis­(2,6- dimethyl-*N*-oxidoformamidinium di­chloro­methane solvate (CSD Refcode QUCMUX; Cibian *et al.*, 2009 ▸).

**Figure 2:**
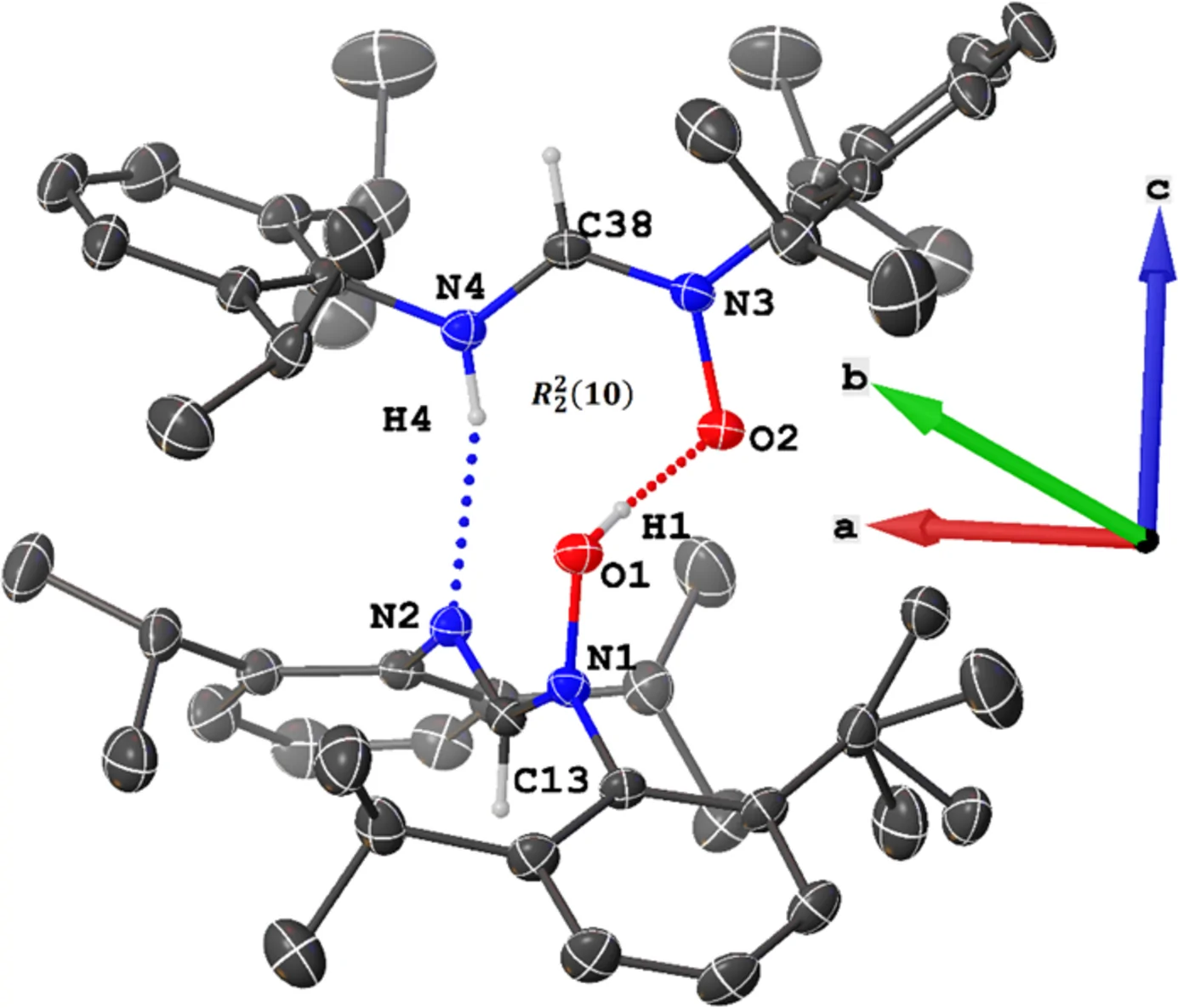
Illustration of one of the two O—H⋯O and N—H⋯N hydrogen-bonding patterns in the crystal of the title compound (red and blue dotted bonds, respectively).

The mol­ecular packing of the title compounds is consolidated by O—H⋯O and N—H⋯N hydrogen-bonding inter­actions (Table 1 ▸; Fig. 2 ▸). These inter­actions occur between the zwitterionic and neutral mol­ecules, giving rise to hydrogen-bonded cyclic dimers.

## Synthesis and crystallization

The title compound was synthesized following a literature procedure (Juma *et al.*, 2025 ▸). The crude solid was then recrystallized from its di­chloro­methane solution to produce colourless blocks of the title compound suitable for X-ray diffraction.

## Refinement

Crystallographic data and structure refinement details are summarized in Table 2 ▸. The hydrogen atoms on the formamidine backbone and the disordered terminal carbon atoms of the C11-isopropyl substituent were refined as disordered over two positions of equal site occupancy. All disordered bond lengths and bond angles were restrained to be similar to each other (SADI restraints, s.d. 0.02 Å), and *U^ij^* components of the ADPs of the disordered atoms closer to each other than 2.0 Å were restrained to be similar (SIMU restraint, s.d. 0.01 Å).

## Supplementary Material

Crystal structure: contains datablock(s) I. DOI: 10.1107/S2414314626008059/tk4127sup1.cif

Structure factors: contains datablock(s) I. DOI: 10.1107/S2414314626008059/tk4127Isup3.hkl

CCDC reference: 2578952

Additional supporting information:  crystallographic information; 3D view; checkCIF report

## Figures and Tables

**Table 1:** Hydrogen-bond geometry (Å, °)

*D*—H⋯*A*	*D*—H	H⋯*A*	*D*⋯*A*	*D*—H⋯*A*
O1—H1⋯O2	0.82	1.76	2.577 (3)	175
N2—H2⋯N4	0.86	2.09	2.906 (3)	158
O2—H2*A*⋯O1	0.82	1.76	2.577 (3)	177
N4—H4*A*⋯N2	0.86	2.07	2.906 (3)	164

**Table 2:** Experimental details

Crystal data	
Chemical formula	C_25_H_36_N_2_O
*M*_r_	380.56
Crystal system, space group	Triclinic, *P* 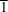
Temperature (K)	293
*a*, *b*, *c* (Å)	13.019 (4), 14.090 (5), 14.497 (5)
α, β, γ (°)	83.167 (7), 73.864 (8), 70.987 (8)
*V* (Å^3^)	2413.9 (13)
*Z*	4
Radiation type	Mo *K*α
μ (mm^−1^)	0.06
Crystal size (mm)	0.28 × 0.22 × 0.18

Data collection	
Diffractometer	Bruker APEXII CCD
Absorption correction	Multi-scan (*SADABS*; Krause *et al.*, 2015 ▸)
*T*_min_, *T*_max_	0.641, 0.745
No. of measured, independent and observed [*I* > 2σ(*I*)] reflections	25947, 9358, 5940
*R*_int_	0.037
(sin θ/λ)_max_ (Å^−1^)	0.618

Refinement	
*R*[*F*^2^ > 2σ(*F*^2^)], *wR*(*F*^2^), *S*	0.080, 0.184, 1.14
No. of reflections	9358
No. of parameters	529
No. of restraints	2
H-atom treatment	H-atom parameters constrained
Δρ_max_, Δρ_min_ (e Å^−3^)	0.22, −0.17

Computer programs: *APEX2* and *SAINT* (Bruker, 2009 ▸), *SHELXS* (Sheldrick, 2008 ▸), *SHELXL2018/3* (Sheldrick, 2015 ▸) and *OLEX2* (Dolomanov *et al.*, 2009 ▸).

## full crystallographic data

### N,N'-Bis(2,6-diisopropylphenyl)-N-hydroxyformamidine–N,N'-bis(2,6-diisopropylphenyl)-N-oxidoformamidinium (1/1) Crystal data

**Table:**

C_25_H_36_N_2_O	*Z* = 4
*M**_r_* = 380.56	*F*(000) = 832
Triclinic, *P*1	*D*_x_ = 1.047 Mg m^−^^3^
*a* = 13.019 (4) Å	Mo *K*α radiation, λ = 0.71073 Å
*b* = 14.090 (5) Å	Cell parameters from 8283 reflections
*c* = 14.497 (5) Å	θ = 3.3–26.6°
α = 83.167 (7)°	µ = 0.06 mm^−^^1^
β = 73.864 (8)°	*T* = 293 K
γ = 70.987 (8)°	Block, colourless
*V* = 2413.9 (13) Å^3^	0.28 × 0.22 × 0.18 mm

### N,N'-Bis(2,6-diisopropylphenyl)-N-hydroxyformamidine–N,N'-bis(2,6-diisopropylphenyl)-N-oxidoformamidinium (1/1) Data collection

**Table:**

Bruker APEXII CCD diffractometer	5940 reflections with *I* > 2σ(*I*)
Graphite monochromator	*R*_int_ = 0.037
φ and ω scans	θ_max_ = 26.1°, θ_min_ = 1.5°
Absorption correction: multi-scan (SADABS; Krause *et al.*, 2015)	*h* = −14→16
*T*_min_ = 0.641, *T*_max_ = 0.745	*k* = −17→16
25947 measured reflections	*l* = −17→15
9358 independent reflections	

### N,N'-Bis(2,6-diisopropylphenyl)-N-hydroxyformamidine–N,N'-bis(2,6-diisopropylphenyl)-N-oxidoformamidinium (1/1) Refinement

**Table:**

Refinement on *F*^2^	Primary atom site location: structure-invariant direct methods
Least-squares matrix: full	Hydrogen site location: inferred from neighbouring sites
*R*[*F*^2^ > 2σ(*F*^2^)] = 0.080	H-atom parameters constrained
*wR*(*F*^2^) = 0.184	*w* = 1/[σ^2^(*F*_o_^2^) + (0.0499*P*)^2^ + 0.9506*P*] where *P* = (*F*_o_^2^ + 2*F*_c_^2^)/3
*S* = 1.14	(Δ/σ)_max_ < 0.001
9358 reflections	Δρ_max_ = 0.22 e Å^−^^3^
529 parameters	Δρ_min_ = −0.16 e Å^−^^3^
2 restraints	

### N,N'-Bis(2,6-diisopropylphenyl)-N-hydroxyformamidine–N,N'-bis(2,6-diisopropylphenyl)-N-oxidoformamidinium (1/1) Special details

**Table:**

Geometry. All esds (except the esd in the dihedral angle between two l.s. planes) are estimated using the full covariance matrix. The cell esds are taken into account individually in the estimation of esds in distances, angles and torsion angles; correlations between esds in cell parameters are only used when they are defined by crystal symmetry. An approximate (isotropic) treatment of cell esds is used for estimating esds involving l.s. planes.

### N,N'-Bis(2,6-diisopropylphenyl)-N-hydroxyformamidine–N,N'-bis(2,6-diisopropylphenyl)-N-oxidoformamidinium (1/1) Fractional atomic coordinates and isotropic or equivalent isotropic displacement parameters (Å^2^)

**Table:**

	*x*	*y*	*z*	*U*_iso_*/*U*_eq_	Occ. (<1)
O1	0.12858 (15)	0.81337 (13)	0.17059 (11)	0.0580 (5)	
H1	0.125355	0.778756	0.220332	0.087*	0.5
N1	0.19331 (17)	0.75397 (15)	0.09407 (13)	0.0499 (5)	
N2	0.35095 (16)	0.70872 (15)	0.15412 (14)	0.0494 (5)	
H2	0.317994	0.750145	0.200100	0.059*	0.5
C1	0.1393 (2)	0.75386 (19)	0.01951 (17)	0.0522 (6)	
C2	0.1564 (3)	0.8159 (2)	−0.0625 (2)	0.0687 (8)	
C3	0.1032 (3)	0.8119 (3)	−0.1328 (2)	0.0885 (10)	
H3	0.114871	0.849987	−0.189470	0.106*	
C4	0.0344 (3)	0.7535 (3)	−0.1205 (2)	0.0890 (11)	
H4	−0.000366	0.752889	−0.168362	0.107*	
C5	0.0165 (3)	0.6960 (2)	−0.0385 (2)	0.0764 (9)	
H5	−0.031595	0.657577	−0.030855	0.092*	
C6	0.0690 (2)	0.6937 (2)	0.03415 (19)	0.0590 (7)	
C7	0.3214 (3)	0.8676 (3)	−0.1672 (3)	0.1215 (14)	
H7A	0.363457	0.913710	−0.172974	0.182*	
H7B	0.370071	0.800031	−0.164824	0.182*	
H7C	0.290376	0.876791	−0.221678	0.182*	
C8	0.2261 (3)	0.8875 (3)	−0.0751 (2)	0.0838 (10)	
H8	0.260108	0.875317	−0.020662	0.101*	
C9	0.1518 (4)	0.9972 (3)	−0.0731 (3)	0.1234 (15)	
H9A	0.197479	1.040584	−0.081143	0.185*	
H9B	0.115056	1.010474	−0.124363	0.185*	
H9C	0.096374	1.009389	−0.012696	0.185*	
C10	0.0541 (9)	0.5239 (7)	0.1096 (10)	0.095 (3)	0.5
H10A	−0.009425	0.525155	0.087658	0.143*	0.5
H10B	0.053531	0.485742	0.169053	0.143*	0.5
H10C	0.122099	0.493434	0.062335	0.143*	0.5
C11	0.0482 (3)	0.6307 (2)	0.1249 (2)	0.0724 (8)	
H11	0.107407	0.625575	0.156397	0.087*	0.5
H11A	0.070679	0.654315	0.174838	0.087*	0.5
C12	−0.0645 (7)	0.6848 (7)	0.1944 (6)	0.090 (2)	0.5
H12A	−0.063564	0.749432	0.208082	0.135*	0.5
H12B	−0.075205	0.645416	0.253000	0.135*	0.5
H12C	−0.124859	0.693091	0.165264	0.135*	0.5
C13	0.3001 (2)	0.70519 (18)	0.08931 (17)	0.0488 (6)	
H13	0.341685	0.665813	0.036355	0.059*	
C14	0.4629 (2)	0.6423 (2)	0.14744 (17)	0.0535 (6)	
C15	0.5525 (2)	0.6832 (2)	0.1227 (2)	0.0659 (8)	
C16	0.6601 (3)	0.6175 (3)	0.1172 (3)	0.0897 (10)	
H16	0.720808	0.642658	0.099978	0.108*	
C17	0.6787 (3)	0.5167 (3)	0.1366 (3)	0.0975 (12)	
H17	0.751488	0.474213	0.131609	0.117*	
C18	0.5902 (3)	0.4782 (3)	0.1634 (2)	0.0861 (10)	
H18	0.603880	0.409738	0.177193	0.103*	
C19	0.4798 (2)	0.5397 (2)	0.17050 (19)	0.0630 (7)	
C20	0.3929 (4)	0.4128 (4)	0.1431 (4)	0.1463 (19)	
H20A	0.401258	0.436765	0.077401	0.219*	
H20B	0.457353	0.356792	0.148071	0.219*	
H20C	0.326509	0.392146	0.164285	0.219*	
C21	0.3827 (3)	0.4963 (2)	0.2054 (2)	0.0750 (8)	
H21	0.314079	0.550618	0.201280	0.090*	
C22	0.3693 (4)	0.4620 (4)	0.3099 (3)	0.1383 (17)	
H22A	0.307508	0.434871	0.330424	0.208*	
H22B	0.436955	0.411209	0.317197	0.208*	
H22C	0.354952	0.518062	0.348423	0.208*	
C23	0.5315 (4)	0.8187 (3)	−0.0061 (3)	0.1116 (13)	
H23A	0.518531	0.889513	−0.019185	0.167*	
H23B	0.602723	0.782373	−0.046206	0.167*	
H23C	0.472913	0.799383	−0.019275	0.167*	
C24	0.5321 (3)	0.7948 (2)	0.0996 (3)	0.0800 (9)	
H24	0.456522	0.829341	0.137708	0.096*	
C25	0.6132 (3)	0.8381 (3)	0.1269 (3)	0.1253 (16)	
H25A	0.595304	0.908557	0.110193	0.188*	
H25B	0.606257	0.828560	0.194767	0.188*	
H25C	0.688916	0.804302	0.092764	0.188*	
O2	0.12206 (17)	0.69493 (13)	0.32048 (12)	0.0628 (5)	
H2A	0.126727	0.732394	0.272711	0.094*	0.5
N3	0.09872 (18)	0.74841 (15)	0.40070 (14)	0.0536 (5)	
N4	0.25744 (17)	0.79968 (15)	0.34108 (14)	0.0523 (5)	
H4A	0.277777	0.764470	0.290847	0.063*	0.5
C26	0.0044 (2)	0.73872 (19)	0.47751 (18)	0.0588 (7)	
C27	0.0237 (3)	0.6675 (2)	0.5517 (2)	0.0798 (9)	
C28	−0.0700 (4)	0.6619 (3)	0.6251 (2)	0.1081 (14)	
H28	−0.060082	0.616607	0.676413	0.130*	
C29	−0.1761 (4)	0.7217 (3)	0.6233 (3)	0.1133 (15)	
H29	−0.237022	0.716933	0.673469	0.136*	
C30	−0.1931 (3)	0.7884 (3)	0.5480 (3)	0.0917 (11)	
H30	−0.265845	0.827380	0.547238	0.110*	
C31	−0.1041 (2)	0.7988 (2)	0.4731 (2)	0.0670 (8)	
C32	−0.2233 (4)	0.8733 (5)	0.3569 (4)	0.166 (2)	
H32A	−0.233890	0.922748	0.305955	0.249*	
H32B	−0.208240	0.807910	0.333534	0.249*	
H32C	−0.290054	0.887738	0.408876	0.249*	
C33	−0.1241 (3)	0.8757 (2)	0.3925 (2)	0.0762 (9)	
H33	−0.056541	0.860448	0.338921	0.091*	
C34	−0.1470 (4)	0.9808 (3)	0.4260 (3)	0.1162 (14)	
H34A	−0.159589	1.028789	0.374228	0.174*	
H34B	−0.212393	0.996243	0.479230	0.174*	
H34C	−0.083505	0.983743	0.445580	0.174*	
C35	0.1436 (4)	0.4870 (3)	0.5446 (4)	0.1419 (18)	
H35A	0.217208	0.442856	0.545429	0.213*	
H35B	0.088973	0.469784	0.597668	0.213*	
H35C	0.127340	0.480393	0.485447	0.213*	
C36	0.1394 (3)	0.5961 (3)	0.5529 (2)	0.0969 (11)	
H36	0.193315	0.612588	0.496321	0.116*	
C37	0.1753 (5)	0.6072 (4)	0.6415 (3)	0.164 (2)	
H37A	0.248604	0.560783	0.639311	0.246*	
H37B	0.177255	0.674543	0.642794	0.246*	
H37C	0.122534	0.593318	0.698252	0.246*	
C38	0.1674 (2)	0.79680 (18)	0.40791 (17)	0.0526 (6)	
H38	0.150654	0.830906	0.463965	0.063*	
C39	0.3230 (2)	0.8611 (2)	0.35050 (19)	0.0577 (7)	
C40	0.3113 (2)	0.9527 (2)	0.2983 (2)	0.0637 (7)	
C41	0.3783 (3)	1.0100 (3)	0.3047 (3)	0.0852 (10)	
H41	0.372364	1.070887	0.270461	0.102*	
C42	0.4536 (3)	0.9773 (3)	0.3615 (3)	0.0998 (13)	
H42	0.497081	1.016686	0.365873	0.120*	
C43	0.4644 (3)	0.8878 (3)	0.4110 (3)	0.0986 (12)	
H43	0.515726	0.866924	0.448442	0.118*	
C44	0.4005 (2)	0.8264 (2)	0.4071 (2)	0.0743 (9)	
C45	0.5297 (4)	0.6509 (3)	0.4136 (4)	0.159 (2)	
H45A	0.538647	0.587766	0.448775	0.239*	
H45B	0.531342	0.641543	0.348662	0.239*	
H45C	0.589676	0.676315	0.413742	0.239*	
C46	0.4179 (3)	0.7253 (3)	0.4607 (3)	0.0972 (11)	
H46	0.358446	0.698818	0.456783	0.117*	
C47	0.4083 (6)	0.7339 (4)	0.5670 (4)	0.190 (3)	
H47A	0.337286	0.781157	0.595538	0.284*	
H47B	0.413569	0.669412	0.598938	0.284*	
H47C	0.468015	0.756490	0.573266	0.284*	
C48	0.2590 (4)	1.0519 (3)	0.1495 (3)	0.1172 (14)	
H48A	0.331620	1.015363	0.111588	0.176*	
H48B	0.204839	1.065944	0.112362	0.176*	
H48C	0.262083	1.113891	0.168108	0.176*	
C49	0.2251 (3)	0.9893 (2)	0.2389 (2)	0.0772 (9)	
H49	0.217365	0.929431	0.216995	0.093*	
C50	0.1114 (3)	1.0455 (3)	0.2993 (3)	0.1122 (14)	
H50A	0.090827	1.005404	0.355414	0.168*	
H50B	0.113973	1.107501	0.318321	0.168*	
H50C	0.056729	1.059554	0.262575	0.168*	
C10A	0.1195 (9)	0.5201 (8)	0.1035 (11)	0.121 (4)	0.5
H10D	0.095814	0.496186	0.056068	0.181*	0.5
H10E	0.109561	0.479573	0.161391	0.181*	0.5
H10F	0.197343	0.516012	0.079697	0.181*	0.5
C12A	−0.0750 (9)	0.6326 (11)	0.1618 (9)	0.155 (5)	0.5
H12D	−0.122619	0.700892	0.169553	0.232*	0.5
H12E	−0.085150	0.596623	0.222468	0.232*	0.5
H12F	−0.094253	0.601425	0.116327	0.232*	0.5

### N,N'-Bis(2,6-diisopropylphenyl)-N-hydroxyformamidine–N,N'-bis(2,6-diisopropylphenyl)-N-oxidoformamidinium (1/1) Atomic displacement parameters (Å^2^)

**Table:**

	*U*^11^	*U*^22^	*U*^33^	*U*^12^	*U*^13^	*U*^23^
O1	0.0547 (11)	0.0666 (11)	0.0509 (10)	−0.0126 (9)	−0.0151 (9)	−0.0088 (8)
N1	0.0474 (12)	0.0596 (12)	0.0424 (11)	−0.0151 (10)	−0.0124 (9)	−0.0014 (9)
N2	0.0442 (11)	0.0553 (12)	0.0499 (11)	−0.0135 (9)	−0.0144 (9)	−0.0053 (9)
C1	0.0530 (15)	0.0566 (15)	0.0486 (14)	−0.0113 (12)	−0.0221 (12)	−0.0015 (12)
C2	0.078 (2)	0.0735 (19)	0.0584 (17)	−0.0207 (16)	−0.0303 (15)	0.0058 (15)
C3	0.116 (3)	0.095 (2)	0.067 (2)	−0.034 (2)	−0.049 (2)	0.0177 (18)
C4	0.118 (3)	0.088 (2)	0.079 (2)	−0.025 (2)	−0.065 (2)	0.0017 (19)
C5	0.085 (2)	0.0711 (19)	0.089 (2)	−0.0242 (17)	−0.0473 (19)	−0.0034 (17)
C6	0.0625 (17)	0.0603 (16)	0.0607 (16)	−0.0159 (14)	−0.0293 (14)	−0.0028 (13)
C7	0.106 (3)	0.130 (3)	0.113 (3)	−0.038 (3)	−0.009 (3)	0.018 (3)
C8	0.096 (3)	0.096 (2)	0.0690 (19)	−0.044 (2)	−0.0319 (18)	0.0290 (18)
C9	0.129 (4)	0.091 (3)	0.152 (4)	−0.047 (3)	−0.023 (3)	−0.001 (3)
C10	0.124 (9)	0.087 (6)	0.093 (6)	−0.059 (6)	−0.030 (7)	0.008 (4)
C11	0.081 (2)	0.083 (2)	0.0742 (19)	−0.0446 (18)	−0.0325 (17)	0.0076 (17)
C12	0.085 (6)	0.127 (7)	0.075 (5)	−0.056 (5)	−0.017 (4)	−0.007 (4)
C13	0.0483 (15)	0.0532 (14)	0.0444 (13)	−0.0179 (12)	−0.0079 (11)	−0.0017 (11)
C14	0.0444 (15)	0.0645 (16)	0.0499 (14)	−0.0101 (12)	−0.0144 (11)	−0.0080 (12)
C15	0.0477 (16)	0.0784 (19)	0.0719 (18)	−0.0175 (14)	−0.0123 (14)	−0.0158 (15)
C16	0.0489 (18)	0.106 (3)	0.113 (3)	−0.0171 (18)	−0.0178 (18)	−0.023 (2)
C17	0.056 (2)	0.105 (3)	0.114 (3)	0.009 (2)	−0.029 (2)	−0.012 (2)
C18	0.076 (2)	0.075 (2)	0.092 (2)	−0.0003 (18)	−0.0253 (19)	−0.0003 (18)
C19	0.0589 (17)	0.0642 (17)	0.0585 (16)	−0.0073 (14)	−0.0172 (13)	−0.0012 (13)
C20	0.161 (5)	0.151 (4)	0.148 (4)	−0.083 (4)	−0.015 (3)	−0.044 (3)
C21	0.079 (2)	0.0600 (17)	0.081 (2)	−0.0184 (16)	−0.0209 (17)	0.0085 (16)
C22	0.160 (4)	0.172 (5)	0.098 (3)	−0.089 (4)	−0.029 (3)	0.039 (3)
C23	0.127 (4)	0.092 (3)	0.119 (3)	−0.051 (2)	−0.021 (3)	0.009 (2)
C24	0.0565 (18)	0.080 (2)	0.105 (3)	−0.0300 (16)	−0.0039 (17)	−0.0223 (19)
C25	0.094 (3)	0.123 (3)	0.180 (4)	−0.056 (3)	−0.022 (3)	−0.045 (3)
O2	0.0705 (12)	0.0709 (12)	0.0478 (10)	−0.0265 (10)	−0.0076 (9)	−0.0091 (9)
N3	0.0563 (13)	0.0590 (13)	0.0436 (11)	−0.0176 (11)	−0.0089 (10)	−0.0036 (10)
N4	0.0505 (13)	0.0568 (12)	0.0497 (11)	−0.0134 (10)	−0.0157 (10)	−0.0037 (10)
C26	0.0686 (18)	0.0524 (15)	0.0500 (14)	−0.0218 (14)	−0.0015 (13)	−0.0044 (12)
C27	0.100 (3)	0.0618 (18)	0.0597 (17)	−0.0181 (17)	0.0001 (17)	0.0000 (15)
C28	0.142 (4)	0.072 (2)	0.072 (2)	−0.027 (2)	0.020 (2)	0.0108 (18)
C29	0.113 (3)	0.079 (2)	0.106 (3)	−0.038 (2)	0.049 (2)	−0.008 (2)
C30	0.074 (2)	0.076 (2)	0.101 (3)	−0.0251 (18)	0.0218 (19)	−0.012 (2)
C31	0.0642 (19)	0.0595 (17)	0.0709 (18)	−0.0225 (14)	0.0013 (15)	−0.0119 (14)
C32	0.138 (4)	0.218 (6)	0.183 (5)	−0.080 (4)	−0.096 (4)	0.045 (4)
C33	0.0566 (18)	0.083 (2)	0.079 (2)	−0.0134 (16)	−0.0134 (15)	0.0004 (17)
C34	0.128 (3)	0.075 (2)	0.113 (3)	−0.016 (2)	−0.002 (3)	0.012 (2)
C35	0.164 (5)	0.078 (3)	0.148 (4)	0.002 (3)	−0.033 (3)	0.003 (3)
C36	0.120 (3)	0.081 (2)	0.067 (2)	−0.010 (2)	−0.020 (2)	0.0134 (17)
C37	0.189 (5)	0.184 (5)	0.105 (3)	−0.015 (4)	−0.065 (4)	−0.004 (3)
C38	0.0579 (16)	0.0536 (15)	0.0446 (13)	−0.0102 (13)	−0.0185 (12)	−0.0017 (11)
C39	0.0467 (15)	0.0641 (17)	0.0626 (16)	−0.0131 (13)	−0.0133 (13)	−0.0151 (14)
C40	0.0568 (17)	0.0624 (17)	0.0743 (18)	−0.0198 (14)	−0.0147 (14)	−0.0120 (15)
C41	0.071 (2)	0.077 (2)	0.111 (3)	−0.0306 (18)	−0.011 (2)	−0.0219 (19)
C42	0.061 (2)	0.110 (3)	0.141 (3)	−0.034 (2)	−0.019 (2)	−0.046 (3)
C43	0.060 (2)	0.118 (3)	0.127 (3)	−0.017 (2)	−0.040 (2)	−0.030 (3)
C44	0.0511 (17)	0.090 (2)	0.085 (2)	−0.0122 (16)	−0.0262 (16)	−0.0201 (18)
C45	0.145 (5)	0.107 (3)	0.189 (5)	0.010 (3)	−0.040 (4)	−0.011 (3)
C46	0.085 (3)	0.106 (3)	0.109 (3)	−0.013 (2)	−0.061 (2)	0.004 (2)
C47	0.260 (7)	0.170 (5)	0.121 (4)	−0.007 (5)	−0.097 (5)	0.008 (4)
C48	0.129 (4)	0.104 (3)	0.099 (3)	−0.026 (3)	−0.019 (2)	0.020 (2)
C49	0.098 (3)	0.0602 (17)	0.090 (2)	−0.0362 (17)	−0.041 (2)	0.0122 (16)
C50	0.066 (2)	0.146 (4)	0.118 (3)	−0.026 (2)	−0.038 (2)	0.038 (3)
C10A	0.145 (11)	0.103 (7)	0.129 (8)	−0.065 (8)	−0.046 (10)	0.043 (6)
C12A	0.110 (8)	0.236 (17)	0.133 (10)	−0.085 (10)	−0.033 (7)	0.035 (10)

### N,N'-Bis(2,6-diisopropylphenyl)-N-hydroxyformamidine–N,N'-bis(2,6-diisopropylphenyl)-N-oxidoformamidinium (1/1) Geometric parameters (Å, º)

**Table:**

O1—H1	0.8200	N3—C26	1.442 (3)
O1—N1	1.372 (2)	N3—C38	1.320 (3)
N1—C1	1.444 (3)	N4—H4A	0.8600
N1—C13	1.321 (3)	N4—C38	1.306 (3)
N2—H2	0.8600	N4—C39	1.439 (3)
N2—C13	1.305 (3)	C26—C27	1.398 (4)
N2—C14	1.436 (3)	C26—C31	1.405 (4)
C1—C2	1.400 (4)	C27—C28	1.395 (5)
C1—C6	1.398 (4)	C27—C36	1.518 (5)
C2—C3	1.398 (4)	C28—H28	0.9300
C2—C8	1.527 (4)	C28—C29	1.370 (6)
C3—H3	0.9300	C29—H29	0.9300
C3—C4	1.366 (5)	C29—C30	1.370 (5)
C4—H4	0.9300	C30—H30	0.9300
C4—C5	1.365 (4)	C30—C31	1.385 (4)
C5—H5	0.9300	C31—C33	1.513 (4)
C5—C6	1.399 (4)	C32—H32A	0.9600
C6—C11	1.508 (4)	C32—H32B	0.9600
C7—H7A	0.9600	C32—H32C	0.9600
C7—H7B	0.9600	C32—C33	1.529 (5)
C7—H7C	0.9600	C33—H33	0.9800
C7—C8	1.531 (5)	C33—C34	1.524 (5)
C8—H8	0.9800	C34—H34A	0.9600
C8—C9	1.533 (5)	C34—H34B	0.9600
C9—H9A	0.9600	C34—H34C	0.9600
C9—H9B	0.9600	C35—H35A	0.9600
C9—H9C	0.9600	C35—H35B	0.9600
C10—H10A	0.9600	C35—H35C	0.9600
C10—H10B	0.9600	C35—C36	1.537 (5)
C10—H10C	0.9600	C36—H36	0.9800
C10—C11	1.520 (9)	C36—C37	1.524 (5)
C11—H11	0.9800	C37—H37A	0.9600
C11—H11A	0.9800	C37—H37B	0.9600
C11—C12	1.543 (8)	C37—H37C	0.9600
C11—C10A	1.548 (11)	C38—H38	0.9300
C11—C12A	1.536 (9)	C39—C40	1.402 (4)
C12—H12A	0.9600	C39—C44	1.405 (4)
C12—H12B	0.9600	C40—C41	1.394 (4)
C12—H12C	0.9600	C40—C49	1.524 (4)
C13—H13	0.9300	C41—H41	0.9300
C14—C15	1.406 (4)	C41—C42	1.384 (5)
C14—C19	1.403 (4)	C42—H42	0.9300
C15—C16	1.392 (4)	C42—C43	1.360 (5)
C15—C24	1.519 (4)	C43—H43	0.9300
C16—H16	0.9300	C43—C44	1.396 (5)
C16—C17	1.368 (5)	C44—C46	1.520 (5)
C17—H17	0.9300	C45—H45A	0.9600
C17—C18	1.371 (5)	C45—H45B	0.9600
C18—H18	0.9300	C45—H45C	0.9600
C18—C19	1.397 (4)	C45—C46	1.518 (5)
C19—C21	1.517 (4)	C46—H46	0.9800
C20—H20A	0.9600	C46—C47	1.526 (6)
C20—H20B	0.9600	C47—H47A	0.9600
C20—H20C	0.9600	C47—H47B	0.9600
C20—C21	1.517 (5)	C47—H47C	0.9600
C21—H21	0.9800	C48—H48A	0.9600
C21—C22	1.515 (5)	C48—H48B	0.9600
C22—H22A	0.9600	C48—H48C	0.9600
C22—H22B	0.9600	C48—C49	1.522 (5)
C22—H22C	0.9600	C49—H49	0.9800
C23—H23A	0.9600	C49—C50	1.505 (5)
C23—H23B	0.9600	C50—H50A	0.9600
C23—H23C	0.9600	C50—H50B	0.9600
C23—C24	1.531 (5)	C50—H50C	0.9600
C24—H24	0.9800	C10A—H10D	0.9600
C24—C25	1.535 (4)	C10A—H10E	0.9600
C25—H25A	0.9600	C10A—H10F	0.9600
C25—H25B	0.9600	C12A—H12D	0.9600
C25—H25C	0.9600	C12A—H12E	0.9600
O2—H2A	0.8200	C12A—H12F	0.9600
O2—N3	1.370 (2)		
			
N1—O1—H1	109.5	C38—N3—C26	123.7 (2)
O1—N1—C1	116.06 (19)	C38—N4—H4A	119.6
C13—N1—O1	120.08 (19)	C38—N4—C39	120.8 (2)
C13—N1—C1	123.8 (2)	C39—N4—H4A	119.6
C13—N2—H2	120.5	C27—C26—N3	119.0 (3)
C13—N2—C14	118.9 (2)	C27—C26—C31	122.4 (3)
C14—N2—H2	120.5	C31—C26—N3	118.6 (2)
C2—C1—N1	119.0 (2)	C26—C27—C36	122.9 (3)
C6—C1—N1	118.1 (2)	C28—C27—C26	116.9 (3)
C6—C1—C2	122.8 (2)	C28—C27—C36	120.2 (3)
C1—C2—C8	122.8 (2)	C27—C28—H28	119.2
C3—C2—C1	116.5 (3)	C29—C28—C27	121.6 (3)
C3—C2—C8	120.6 (3)	C29—C28—H28	119.2
C2—C3—H3	119.1	C28—C29—H29	119.8
C4—C3—C2	121.8 (3)	C28—C29—C30	120.4 (3)
C4—C3—H3	119.1	C30—C29—H29	119.8
C3—C4—H4	119.8	C29—C30—H30	119.3
C5—C4—C3	120.5 (3)	C29—C30—C31	121.3 (4)
C5—C4—H4	119.8	C31—C30—H30	119.3
C4—C5—H5	119.4	C26—C31—C33	122.0 (2)
C4—C5—C6	121.3 (3)	C30—C31—C26	117.4 (3)
C6—C5—H5	119.4	C30—C31—C33	120.5 (3)
C1—C6—C5	117.0 (3)	H32A—C32—H32B	109.5
C1—C6—C11	121.9 (2)	H32A—C32—H32C	109.5
C5—C6—C11	121.0 (3)	H32B—C32—H32C	109.5
H7A—C7—H7B	109.5	C33—C32—H32A	109.5
H7A—C7—H7C	109.5	C33—C32—H32B	109.5
H7B—C7—H7C	109.5	C33—C32—H32C	109.5
C8—C7—H7A	109.5	C31—C33—C32	111.4 (3)
C8—C7—H7B	109.5	C31—C33—H33	108.6
C8—C7—H7C	109.5	C31—C33—C34	110.4 (3)
C2—C8—C7	111.5 (3)	C32—C33—H33	108.6
C2—C8—H8	107.7	C34—C33—C32	109.0 (3)
C2—C8—C9	111.1 (3)	C34—C33—H33	108.6
C7—C8—H8	107.7	C33—C34—H34A	109.5
C7—C8—C9	110.8 (3)	C33—C34—H34B	109.5
C9—C8—H8	107.7	C33—C34—H34C	109.5
C8—C9—H9A	109.5	H34A—C34—H34B	109.5
C8—C9—H9B	109.5	H34A—C34—H34C	109.5
C8—C9—H9C	109.5	H34B—C34—H34C	109.5
H9A—C9—H9B	109.5	H35A—C35—H35B	109.5
H9A—C9—H9C	109.5	H35A—C35—H35C	109.5
H9B—C9—H9C	109.5	H35B—C35—H35C	109.5
H10A—C10—H10B	109.5	C36—C35—H35A	109.5
H10A—C10—H10C	109.5	C36—C35—H35B	109.5
H10B—C10—H10C	109.5	C36—C35—H35C	109.5
C11—C10—H10A	109.5	C27—C36—C35	110.5 (4)
C11—C10—H10B	109.5	C27—C36—H36	107.7
C11—C10—H10C	109.5	C27—C36—C37	112.3 (3)
C6—C11—C10	115.0 (6)	C35—C36—H36	107.7
C6—C11—H11	106.8	C37—C36—C35	110.9 (4)
C6—C11—H11A	109.4	C37—C36—H36	107.7
C6—C11—C12	110.2 (4)	C36—C37—H37A	109.5
C6—C11—C10A	108.6 (6)	C36—C37—H37B	109.5
C6—C11—C12A	112.8 (5)	C36—C37—H37C	109.5
C10—C11—H11	106.8	H37A—C37—H37B	109.5
C10—C11—C12	110.8 (6)	H37A—C37—H37C	109.5
C12—C11—H11	106.8	H37B—C37—H37C	109.5
C10A—C11—H11A	109.4	N3—C38—H38	118.0
C12A—C11—H11A	109.4	N4—C38—N3	123.9 (2)
C12A—C11—C10A	107.1 (7)	N4—C38—H38	118.0
C11—C12—H12A	109.5	C40—C39—N4	118.3 (2)
C11—C12—H12B	109.5	C40—C39—C44	121.7 (3)
C11—C12—H12C	109.5	C44—C39—N4	120.0 (3)
H12A—C12—H12B	109.5	C39—C40—C49	120.5 (2)
H12A—C12—H12C	109.5	C41—C40—C39	118.1 (3)
H12B—C12—H12C	109.5	C41—C40—C49	121.4 (3)
N1—C13—H13	117.9	C40—C41—H41	119.6
N2—C13—N1	124.2 (2)	C42—C41—C40	120.7 (3)
N2—C13—H13	117.9	C42—C41—H41	119.6
C15—C14—N2	118.7 (2)	C41—C42—H42	119.9
C19—C14—N2	119.7 (2)	C43—C42—C41	120.3 (3)
C19—C14—C15	121.5 (2)	C43—C42—H42	119.9
C14—C15—C24	120.7 (2)	C42—C43—H43	119.0
C16—C15—C14	117.7 (3)	C42—C43—C44	121.9 (3)
C16—C15—C24	121.5 (3)	C44—C43—H43	119.0
C15—C16—H16	119.2	C39—C44—C46	122.4 (3)
C17—C16—C15	121.5 (3)	C43—C44—C39	117.3 (3)
C17—C16—H16	119.2	C43—C44—C46	120.3 (3)
C16—C17—H17	119.9	H45A—C45—H45B	109.5
C16—C17—C18	120.2 (3)	H45A—C45—H45C	109.5
C18—C17—H17	119.9	H45B—C45—H45C	109.5
C17—C18—H18	119.3	C46—C45—H45A	109.5
C17—C18—C19	121.4 (3)	C46—C45—H45B	109.5
C19—C18—H18	119.3	C46—C45—H45C	109.5
C14—C19—C21	122.0 (2)	C44—C46—H46	107.5
C18—C19—C14	117.5 (3)	C44—C46—C47	112.4 (4)
C18—C19—C21	120.5 (3)	C45—C46—C44	111.1 (4)
H20A—C20—H20B	109.5	C45—C46—H46	107.5
H20A—C20—H20C	109.5	C45—C46—C47	110.6 (4)
H20B—C20—H20C	109.5	C47—C46—H46	107.5
C21—C20—H20A	109.5	C46—C47—H47A	109.5
C21—C20—H20B	109.5	C46—C47—H47B	109.5
C21—C20—H20C	109.5	C46—C47—H47C	109.5
C19—C21—C20	112.5 (3)	H47A—C47—H47B	109.5
C19—C21—H21	107.2	H47A—C47—H47C	109.5
C20—C21—H21	107.2	H47B—C47—H47C	109.5
C22—C21—C19	110.9 (3)	H48A—C48—H48B	109.5
C22—C21—C20	111.4 (3)	H48A—C48—H48C	109.5
C22—C21—H21	107.2	H48B—C48—H48C	109.5
C21—C22—H22A	109.5	C49—C48—H48A	109.5
C21—C22—H22B	109.5	C49—C48—H48B	109.5
C21—C22—H22C	109.5	C49—C48—H48C	109.5
H22A—C22—H22B	109.5	C40—C49—H49	106.9
H22A—C22—H22C	109.5	C48—C49—C40	114.3 (3)
H22B—C22—H22C	109.5	C48—C49—H49	106.9
H23A—C23—H23B	109.5	C50—C49—C40	111.3 (3)
H23A—C23—H23C	109.5	C50—C49—C48	110.2 (3)
H23B—C23—H23C	109.5	C50—C49—H49	106.9
C24—C23—H23A	109.5	C49—C50—H50A	109.5
C24—C23—H23B	109.5	C49—C50—H50B	109.5
C24—C23—H23C	109.5	C49—C50—H50C	109.5
C15—C24—C23	110.9 (3)	H50A—C50—H50B	109.5
C15—C24—H24	106.8	H50A—C50—H50C	109.5
C15—C24—C25	114.2 (3)	H50B—C50—H50C	109.5
C23—C24—H24	106.8	C11—C10A—H10D	109.5
C23—C24—C25	110.9 (3)	C11—C10A—H10E	109.5
C25—C24—H24	106.8	C11—C10A—H10F	109.5
C24—C25—H25A	109.5	H10D—C10A—H10E	109.5
C24—C25—H25B	109.5	H10D—C10A—H10F	109.5
C24—C25—H25C	109.5	H10E—C10A—H10F	109.5
H25A—C25—H25B	109.5	C11—C12A—H12D	109.5
H25A—C25—H25C	109.5	C11—C12A—H12E	109.5
H25B—C25—H25C	109.5	C11—C12A—H12F	109.5
N3—O2—H2A	109.5	H12D—C12A—H12E	109.5
O2—N3—C26	116.1 (2)	H12D—C12A—H12F	109.5
C38—N3—O2	119.9 (2)	H12E—C12A—H12F	109.5
			
O1—N1—C1—C2	97.6 (3)	C19—C14—C15—C24	−179.0 (3)
O1—N1—C1—C6	−80.3 (3)	C24—C15—C16—C17	−179.0 (3)
O1—N1—C13—N2	0.3 (3)	O2—N3—C26—C27	94.6 (3)
N1—C1—C2—C3	179.3 (3)	O2—N3—C26—C31	−83.1 (3)
N1—C1—C2—C8	−2.6 (4)	O2—N3—C38—N4	1.8 (4)
N1—C1—C6—C5	179.1 (2)	N3—C26—C27—C28	179.1 (3)
N1—C1—C6—C11	0.4 (4)	N3—C26—C27—C36	−3.0 (4)
N2—C14—C15—C16	179.6 (3)	N3—C26—C31—C30	−179.7 (3)
N2—C14—C15—C24	−2.4 (4)	N3—C26—C31—C33	−2.4 (4)
N2—C14—C19—C18	−179.6 (2)	N4—C39—C40—C41	−177.5 (2)
N2—C14—C19—C21	−1.8 (4)	N4—C39—C40—C49	4.3 (4)
C1—N1—C13—N2	176.6 (2)	N4—C39—C44—C43	177.9 (3)
C1—C2—C3—C4	2.5 (5)	N4—C39—C44—C46	−0.6 (4)
C1—C2—C8—C7	123.9 (3)	C26—N3—C38—N4	174.9 (2)
C1—C2—C8—C9	−111.9 (3)	C26—C27—C28—C29	1.6 (5)
C1—C6—C11—C10	−134.9 (5)	C26—C27—C36—C35	−114.4 (4)
C1—C6—C11—C12	99.0 (4)	C26—C27—C36—C37	121.2 (4)
C1—C6—C11—C10A	−102.4 (5)	C26—C31—C33—C32	137.3 (4)
C1—C6—C11—C12A	139.1 (7)	C26—C31—C33—C34	−101.4 (3)
C2—C1—C6—C5	1.3 (4)	C27—C26—C31—C30	2.7 (4)
C2—C1—C6—C11	−177.5 (3)	C27—C26—C31—C33	180.0 (3)
C2—C3—C4—C5	−0.6 (6)	C27—C28—C29—C30	0.7 (6)
C3—C2—C8—C7	−58.0 (4)	C28—C27—C36—C35	63.4 (4)
C3—C2—C8—C9	66.1 (4)	C28—C27—C36—C37	−60.9 (5)
C3—C4—C5—C6	−1.1 (5)	C28—C29—C30—C31	−1.3 (6)
C4—C5—C6—C1	0.8 (4)	C29—C30—C31—C26	−0.3 (5)
C4—C5—C6—C11	179.6 (3)	C29—C30—C31—C33	−177.7 (3)
C5—C6—C11—C10	46.4 (6)	C30—C31—C33—C32	−45.5 (4)
C5—C6—C11—C12	−79.7 (4)	C30—C31—C33—C34	75.8 (4)
C5—C6—C11—C10A	79.0 (6)	C31—C26—C27—C28	−3.3 (4)
C5—C6—C11—C12A	−39.6 (7)	C31—C26—C27—C36	174.6 (3)
C6—C1—C2—C3	−2.9 (4)	C36—C27—C28—C29	−176.4 (4)
C6—C1—C2—C8	175.2 (3)	C38—N3—C26—C27	−78.7 (3)
C8—C2—C3—C4	−175.6 (3)	C38—N3—C26—C31	103.6 (3)
C13—N1—C1—C2	−78.8 (3)	C38—N4—C39—C40	−102.3 (3)
C13—N1—C1—C6	103.3 (3)	C38—N4—C39—C44	80.8 (3)
C13—N2—C14—C15	110.0 (3)	C39—N4—C38—N3	174.6 (2)
C13—N2—C14—C19	−73.3 (3)	C39—C40—C41—C42	−0.4 (4)
C14—N2—C13—N1	171.9 (2)	C39—C40—C49—C48	−148.6 (3)
C14—C15—C16—C17	−1.1 (5)	C39—C40—C49—C50	85.7 (3)
C14—C15—C24—C23	−85.2 (3)	C39—C44—C46—C45	107.7 (4)
C14—C15—C24—C25	148.6 (3)	C39—C44—C46—C47	−127.8 (4)
C14—C19—C21—C20	123.2 (3)	C40—C39—C44—C43	1.2 (4)
C14—C19—C21—C22	−111.2 (3)	C40—C39—C44—C46	−177.3 (3)
C15—C14—C19—C18	−3.1 (4)	C40—C41—C42—C43	0.9 (5)
C15—C14—C19—C21	174.7 (2)	C41—C40—C49—C48	33.2 (4)
C15—C16—C17—C18	−0.9 (6)	C41—C40—C49—C50	−92.5 (4)
C16—C15—C24—C23	92.7 (4)	C41—C42—C43—C44	−0.4 (6)
C16—C15—C24—C25	−33.6 (4)	C42—C43—C44—C39	−0.7 (5)
C16—C17—C18—C19	0.8 (6)	C42—C43—C44—C46	177.9 (3)
C17—C18—C19—C14	1.1 (5)	C43—C44—C46—C45	−70.8 (4)
C17—C18—C19—C21	−176.7 (3)	C43—C44—C46—C47	53.7 (5)
C18—C19—C21—C20	−59.0 (4)	C44—C39—C40—C41	−0.7 (4)
C18—C19—C21—C22	66.5 (4)	C44—C39—C40—C49	−178.9 (3)
C19—C14—C15—C16	3.1 (4)	C49—C40—C41—C42	177.9 (3)

### N,N'-Bis(2,6-diisopropylphenyl)-N-hydroxyformamidine–N,N'-bis(2,6-diisopropylphenyl)-N-oxidoformamidinium (1/1) Hydrogen-bond geometry (Å, º)

**Table:**

*D*—H···*A*	*D*—H	H···*A*	*D*···*A*	*D*—H···*A*
O1—H1···O2	0.82	1.76	2.577 (3)	175
N2—H2···N4	0.86	2.09	2.906 (3)	158
O2—H2*A*···O1	0.82	1.76	2.577 (3)	177
N4—H4*A*···N2	0.86	2.07	2.906 (3)	164
